# Targeting TRPV1 channels: new perspectives on the mechanisms of analgesia for herpes zoster neuralgia and advances in therapeutic research

**DOI:** 10.3389/fcimb.2026.1841790

**Published:** 2026-07-15

**Authors:** Ying Zhang, Yuanyuan Li, Niqin Xiao, Fan Wu

**Affiliations:** 1 The Fourth Affiliated Hospital of Yunnan University of Chinese Medicine, Yuxi, China; 2 Yunnan University of Chinese Medicine, Kunming, China

**Keywords:** immune interactions, neuroinflammation, pain, herpes zoster neuralgia, therapeutic strategies, transient receptor potential vanilloid 1

## Abstract

Herpes Zoster Neuralgia (HZN) is a condition caused by the varicella-zoster virus, characterized primarily by pain. The Transient Receptor Potential Vanilloid 1 (TRPV1), which acts as a pain mediator, plays a significant role in pain signaling and the regulation of neuroinflammation. In this context, this paper summarizes the biological characteristics of TRPV1, its dynamic expression in HZN, and its mechanisms for mediating neuroinflammatory processes and pain signal conduction. It posits that the inhibition of TRPV1 may alleviate HZN. Furthermore, the paper discusses the research and development progress, as well as the challenges associated with TRPV1 antagonists as a new class of analgesics, thereby providing new insights for the treatment of HZN.

## 1 Introduction

Herpes Zoster Neuralgia (HZN) refers to paroxysmal, lightning-like or tingling neural pain that occurs following infection with the Varicella-Zoster Virus (VZV) (Lim et al., 2024; Yue and Yao, 2024). The overall annual incidence of shingles in China is 1.90 to 6.95 cases per thousand individuals. Although the cases occur in all ages, they are mainly concentrated in individuals aged 50 and older. The incidence rate increases with age, and the severity of the disease also worsens with age (Yang et al., 2019; Xia et al., 2025; Zhou et al., 2025). In patients with shingles, factors such as local inflammatory reactions and neuronal damage can trigger the continuous transmission of ectopic discharges and pain signals. This will promote peripheral and central sensitization, leading to the progression of the disease (Liu et al., 2024). However, even if first-line medications such as gabapentin and pregabalin are used appropriately to inhibit central sensitization, their efficacy is still insufficient to completely prevent the chronicization of pain. Consequently, some patients ultimately develop intractable postherpetic neuralgia (PHN) (Shi and Song, 2024). Therefore, identifying highly effective and safe prevention and treatment methods remains a critical issue that requires urgent resolution in clinical practice.

The capsaicin receptor, or Transient Receptor Potential Vanilloid 1 (TRPV1), is a crucial pain-mediating component implicated in several pain pathways. In recent years, research on TRPV1 antagonists as a new generation of analgesic drugs has made significant progress (Gao et al., 2024; González et al., 2025; Zhou and Verne, 2025). The burning sensation in the skin associated with shingles is comparable to the pain generated by the activation of the TRPV1 pathway, underscoring the significant role this channel plays in pain perception (Basbaum et al., 2009; Xia et al., 2011; Iftinca et al., 2021; Li et al., 2024). Furthermore, research indicates that TRPV1 protein and mRNA levels are elevated in the skin lesions of shingles patients, confirming a correlation between TRPV1 and pain experienced by individuals with shingles (Han et al., 2016; Xiao et al., 2023). Importantly, TRPV1 is not merely a peripheral nociceptive receptor but functions as a critical integrative hub that links multiple key mechanisms underlying HZN, including nociceptive signal transduction, neuroinflammation, and neuroimmune interactions. Unlike many single-pathway targets, TRPV1 responds to diverse stimuli such as heat, protons, and inflammatory mediators, and is involved in both peripheral and central sensitization processes (Kim et al., 2014; Maximiano et al., 2024; Liu et al., 2026). This multimodal and cross-system involvement makes TRPV1 a particularly promising and distinctive therapeutic target for HZN. For the purpose of offering new perspectives on the diagnosis and management of HZN, this research will examine the biological traits of TRPV1 and its function in the pathophysiology of HZN.

## 2 An introduction to the TRPV1 channel

The discovery of the TRPV1 channel by David Julius, the 2021 Nobel Prize winner in Physiology or Medicine, has unveiled a critical mechanism through which the body interprets mechanical, thermal, and cold stimuli, converting them into nerve impulses (Julius, 2013; Zhang et al., 2021). As a primary target for analgesic drugs, TRPV1 is widely involved in pain signal transmission and neuroinflammatory regulation (Ji et al., 2016; Pulskamp et al., 2024; Liu et al., 2026). Numerous stimuli, including compounds such as capsaicin, heat exposure (temperature >43 °C), an acidic environment (pH <6.0), and endogenous protons, can activate this channel (Caterina et al., 1997; Bejoma et al., 2026). Upon activation, TRPV1 facilitates the influx of extracellular calcium ions (Ca^2+^.), triggering physiological and pathological reactions, making it one of the key molecules for pain perception (Moran and Szallasi, 2018; Awad-Igbaria et al., 2025; Çiğ, 2025). TRPV1 channels are predominantly located in the sensory neurons of the peripheral nervous system (PNS), such as dorsal root ganglion (DRG) and trigeminal ganglion (TG) (Mezey et al., 2000; Basbaum et al., 2009; Bevan et al., 2014; Fernández-Carvajal et al., 2022). TRPV1 belongs to the TRPV family, exhibits high permeability to Ca^2+^, and is widely distributed in neurons, immune cells, organ epithelial cells and keratinocytes (Caterina et al., 1997; Storozhuk et al., 2019). Its distribution in neurons is mainly concentrated in peripheral injury neurons, and sensory nerve fibers are mainly composed of unmyelin sheath C-type fibers and some thin myelinth Aδ fibers (Guo et al., 1999; Nersesyan et al., 2017; Abbas, 2020). Following the detection of external stimuli, the TRPV1 channel transduces these stimuli into neural signals, relaying them to the central nervous system (CNS), such as the superficial layer of the dorsal angle of the spinal cord and the preoptic area (POA) of the anterior hypothalamus (Nakamura and Morrison, 2008; Kim et al., 2014; Sun et al., 2020). As a result, TRPV1 channels may play a pivotal regulatory role in the onset and progression of neuropathic pain disorders, such as HZN (Badr et al., 2025).

## 3 Dynamic expression of TRPV1 in herpes zoster

### 3.1 VZV infection upregulates TRPV1 expression in DRG neurons

**Figure 1:**
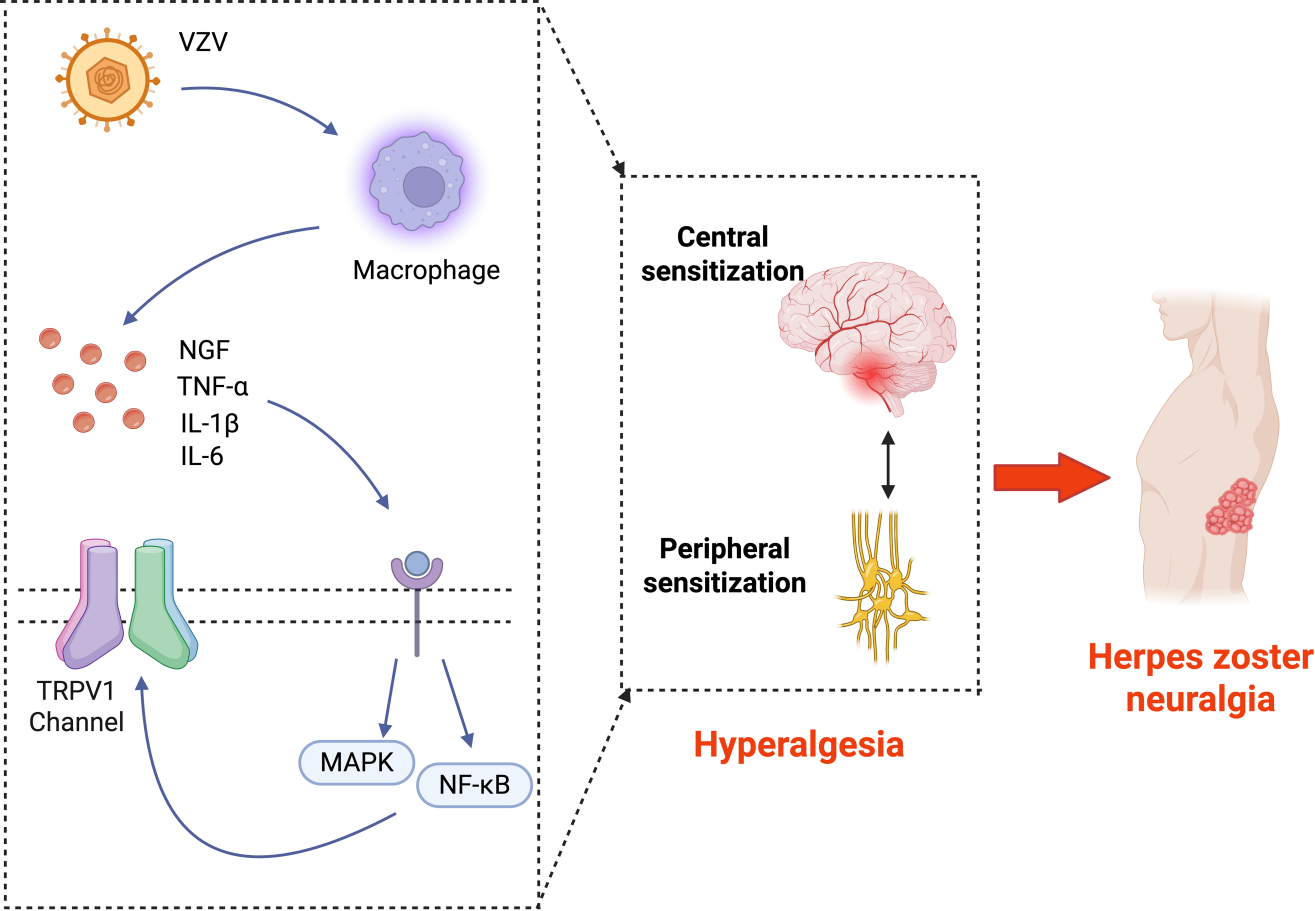
The DRG’s satellite glial cells are infected by VZV. Numerous cytokines and chemokines, including TNF-α, IL-1β, NGF, and others, can be released by activated infected satellite glial cells or infiltrating immune cells (such macrophages). TRPV1 is transcriptionally upregulated and functionally sensitized in neurons as a result of these inflammatory mediators binding to their corresponding receptors on the surface of neurons and activating downstream signaling pathways like the NF-κB and MAPK pathways.

VZV can effectively infect DRGs in both human and animal models, such as SCID-hu mice and rat models, infecting not only neurons but also surrounding satellite glial cells (SGCs) (Warwick and Hanani, 2016; Wu et al., 2023). Research on gene expression profiles shows that VZV infection significantly changes the transcription levels of numerous host genes in DRG. These genes are involved in various pathways, including inflammation, immune response, apoptosis and ion channel function, potentially leading to TRPV1 upregulation (Ouwendijk et al., 2012; Guedon et al., 2015; Pinho-Ribeiro et al., 2017). Li et al. (2021) showed that VZV promotes the expression of the T-type calcium channel Cav3.2 in the dorsal horn of the spinal cord and DRG, which aids in maintaining herpes zoster-related discomfort. VZV not only infects neurons, but also satellite glial cells in DRG (Warwick and Hanani, 2016). Activated infected satellite glial cells or infiltrating immune cells, such as macrophages, can release significant quantities of interleukin-1β (IL-1β), tumor necrosis factor-α (TNF-α), nerve growth factor (NGF), and other cytokines and chemokines (Ji et al., 2018; Gao et al., 2024; Shen and Lin, 2025). Nuclear factor κB (NF-κB) and mitogen-activated protein kinase (MAPK) are examples of downstream signaling pathways that are triggered when these inflammatory mediators bind to their corresponding receptors on neuronal surfaces (Ji and Strichartz, 2004; Gao and Ji, 2010). This activation ultimately leads to the upregulation of transcription and functional sensitization of TRPV1 in neurons. This “bystander effect” exemplifies the classical mechanism of pain sensitization induced by neuroinflammation (Zerboni et al., 2005; Chen et al., 2025a). In conclusion, the current study has confirmed that there is a significant association between VZV infection and the upregulation of TRPV1 expression in DRG neurons, providing an important molecular target for elucidating VZV-related neuropathological pain (**Figure 1**).

### 3.2 Colocation of skin keratin cells and TRPV1 sensory nerve endings

The skin serves not only as a physical barrier but also as a complex sensory organ. Traditionally, sensation was thought to be primarily perceived through nerve endings; however, increasing evidence suggests that epidermal keratinocytes, classified as “non-neuron” cells, play a significant role in sensory transmission (Vidal Yucha et al., 2019; Fernández-Carvajal et al., 2022). Both neurons and epidermal cells express various neurotransmitters and certain receptors that facilitate communication between sensory cells and epidermal cells (Shin et al., 2023). Peier et al. (Peier et al., 2002; Mandadi et al., 2009) demonstrated that thermally stimulated keratinocytes establish a direct link with sensory neurons by secreting adenosine triphosphate (ATP), which conveys temperature information to these neurons. Additionally, keratinocytes release chemicals like NGF and IL-1α to communicate with neurons (Dallos et al., 2006). Although the expression of TRPV1 in skin keratinocytes is low, it is detectable (Denda et al., 2007). TRPV1, a polymodal nociceptor, is essential for transmitting pain and heat sensation signals and is activated by a variety of endogenous and exogenous stimuli (Xiao et al., 2023). The high expression of TRPV1 in primary sensory neurons is tightly linked to its classical function. Primary sensory neurons possess cell bodies located in DRG and TG, with their axon terminals extending to adjacent tissues, including the skin (Zylka et al., 2005; Usoskin et al., 2015). Research indicates that TRPV1 expressed in selectively stimulated keratinocytes is sufficient to activate adjacent nociceptive neurons, thereby inducing acute pain behavior in animal models (Baumbauer et al., 2015; Pang et al., 2015; Talagas et al., 2020). This finding highlights that TRPV1 in keratinoblasts is not only present, but also functionally active, capable of initiating sensory signaling pathways. The colocalization of these keratin-forming cells establishes them as the “first line of defense” within the sensory system. When the skin encounters harmful heat or chemical stimuli, TRPV1 on the keratinocyte membrane is activated, leading to the rapid release of ATP and other signaling molecules into extracellular space. These ATP molecules effectively bind to receptors such as P2X on nerve endings, activating neurons to transmit harmful signals to the brain, resulting in pain or a sensation of heat (Baumbauer et al., 2015; Talagas et al., 2020; Shindo et al., 2021). Consequently, the close physical proximity between TRPV1-expressing sensory nerve endings and keratinocytes, along with the neuro-keratin network of keratinocytes with TRPV1-positive nerve endings, forms a highly efficient functional unit. This unit facilitates effective transmission of chemical or physical signals and is essential for many physiological and pathological processes (Roosterman et al., 2006; Ji et al., 2016). Therefore, the novel perspective of keratinocyte-TRPV1 colocalization at sensory nerve endings warrants further investigation into the epidermal microenvironment of HZN-damaged regions to identify additional therapeutic targets.

## 4 TRPV1-mediated neuroinflammation and pain signal transmission

### 4.1 Inflammatory mediators released by damaged skin sensitize nociceptive neurons via TRPV1

Numerous inflammatory mediators, which involves prostaglandin (PG) and bradykinin (BK), are produced and released at the site of herpes zoster lesions as a result of local peripheral inflammation and neuroinflammation. This process induces neuroinflammation and increases the sensitivity of nociceptors, lowering their activation threshold and causing them to respond with pain to normally innocuous stimuli, such as light touch (Torebjörk et al., 1992; Pinho-Ribeiro et al., 2017; Schumacher, 2024). BK is a potent mediator of inflammation; by binding to B2 receptors, it stimulates cyclooxygenase (COX) and 12-lipoxygenase (12-LOX) to release metabolites that activate TRPV1 (Al-Shamlan and El-Hashim, 2019). Inositol triphosphate (IP3) and diacylglycerol (DAG) are generated by BK through the hydrolysis of 4,5-phosphatidylinositol bisphosphate (PIP2), a process mediated by phospholipase C (PLC). IP3 can stimulate the release of intracellular calcium and activate protein kinase C (PKC) (Grace et al., 2012). PKC activates TRPV1 by phosphorylating specific sites, namely S502, S800, and T704 (Wang et al., 2015). Prostaglandin E2 (PGE2), a crucial member of the prostaglandin family, directly sensitizing nociceptors in DRG neurons via EP(1-4) receptors. Ma et al. (2017) demonstrated that PGE2 enhances the translocation of TRPV1 to the cell surface of DRG neurons through the EP1/CaMKII/PLC/PKC/PKCϵ and EP4/cAMP/PKA/ERK/MAPK signaling pathways. PGE2 stimulates EP4 to bind with Gs protein receptors, leading to an increase in intracellular cyclic adenosine monophosphate (cAMP) levels, which subsequently activates protein kinase A (PKA) to phosphorylate TRPV1 at sites T370 and S116, thereby activating TRPV1 (Kawabata, 2011).

### 4.2 An acidic microenvironment leads to the activation of TRPV1

**Figure 2:**
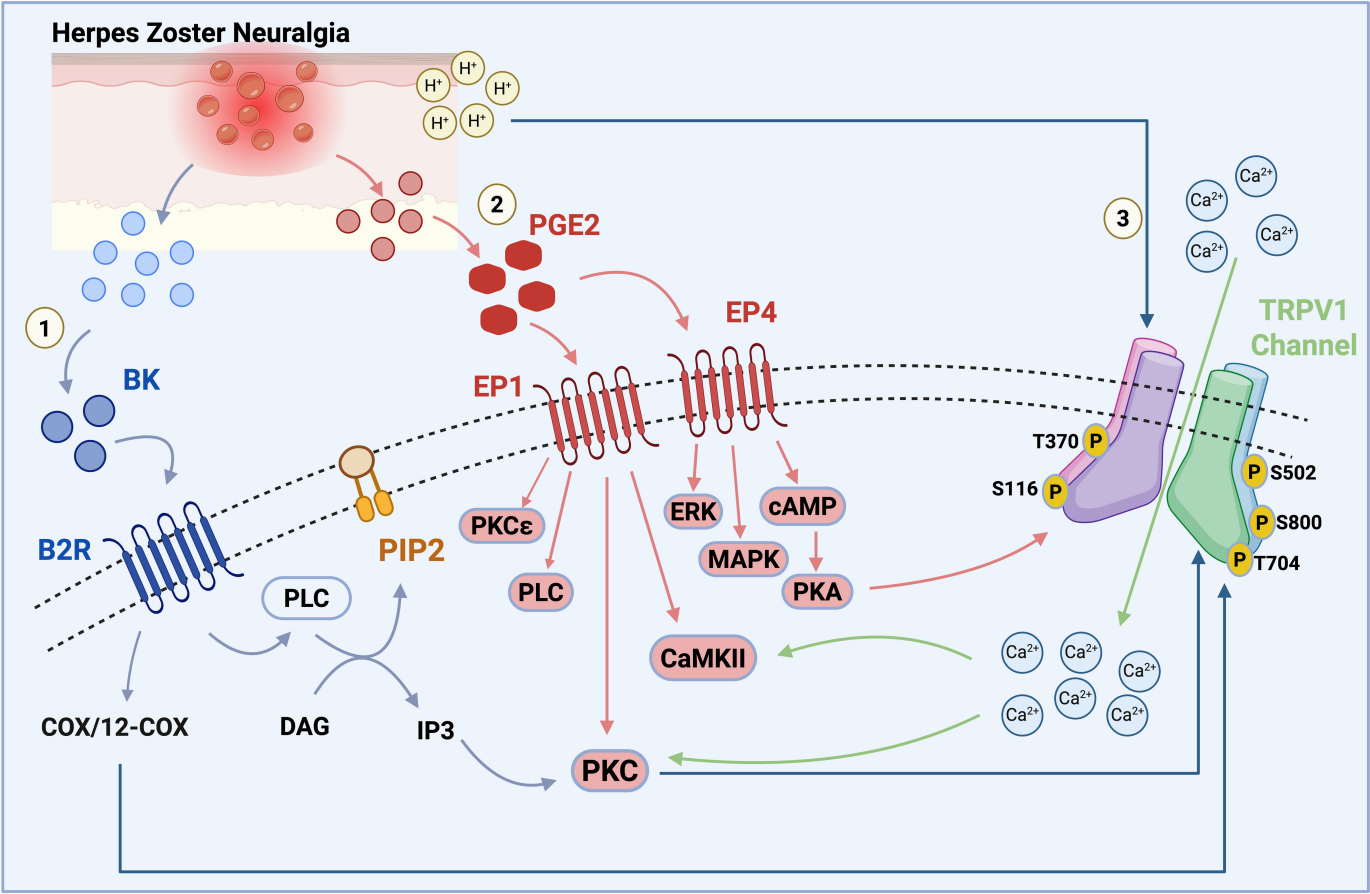
①BK may activate TRPV1 by interacting to the B2 receptor and stimulating the release of metabolites from COX and 12-LOX. Additionally, BK binds to the B2 receptor to produce IP3 and DAG, which triggers PLC-mediated hydrolysis of PIP2. IP3 can activate PKC and encourage intracellular calcium release. By interacting with TRPV1’s S502, S800, and T704 phosphorylation sites, PKC can activate TRPV1. ②Through the EP1/CaMKII/PLC/PKC/PKCϵ and EP4/cAMP/PKA/ERK/MAPK signaling pathways, PGE2 enhances the translocation of TRPV1 to the cell surface of DRG neurons. PGE2 increases cellular cAMP levels, promotes EP4 binding to the Gs protein receptor, and subsequently triggers PKA to act on TRPV1’s T370 and S116 phosphorylation sites, activating TRPV1. ③Herpes zoster lesions have higher local H^+^ concentrations, which lower pH at the inflammatory site. This can directly activate TRPV1 and enhance Ca2^+^ influx. This process will further activate CaMKII and PKC, leading to phosphorylation, promoting the release of related neurotransmitters and neuropeptides, causing peripheral and central sensitization, thereby leading to neuralgia.

The inflammatory reaction leads to a decrease in pH at the inflammatory site due to an increase in H^+^ concentration. This reduction can directly activate TRPV1, resulting in nociceptive nerve impulses being transmitted to the central nervous system, which ultimately leads to the perception of nociceptive pain (Manosalva et al., 2021; Li et al., 2022). Activation of TRPV1 induces an increased influx of Ca^2+^, which subsequently activates Ca^2+^/calmodulin-dependent protein kinase II (CaMKII) and PKC, leading to phosphorylation (Marek-Jozefowicz et al., 2023; Maximiano et al., 2024; Jiang et al., 2026). This cascade promotes the release of various neurotransmitters and neuropeptides, resulting in peripheral and central sensitization, ultimately culminating in neuropathic pain (Quartu et al., 2016; Tang et al., 2025a). Following VZV infection, patients exhibit inflammatory responses at the affected site. Within this inflammatory microenvironment, the enhancement of tissue metabolic activity mainly depends on anaerobic glycolysis to produce energy. This metabolic process generates a substantial amount of acidic metabolites, such as lactic acid and ketones, during the breakdown of sugars and fats, which further decreases the local pH. In the core inflammatory area, the concentration of H^+^ can increase by up to 50 times (Youm et al., 2015; Aki et al., 2020). Consequently, in the acidic inflammatory environment of HZN, TRPV1 is activated, facilitating the influx of Ca^2+^ and subsequently activating CaMKII. This activation triggers the production of numerous inflammatory mediators, leading to peripheral and central sensitization of HZN. This process mediates inflammatory response and pain generation, thus aggravating the neuropathic pain experienced in HZN (**Figure 2**).

## 5 TRPV1 interacts with the immune microenvironment

### 5.1 Mast cell degranulation and the release of histamine and NGF are mediated by TRPV1

Mast cells serve as sentinels for the immune system and are present in various tissues exposed to the external environment, including the skin, respiratory tract, and gastrointestinal mucosa (Paivandy and Pejler, 2021). When stimulated by allergens, pathogens, or physical agents, mast cells undergo rapid degranulation, releasing substantial quantities of pre-stored bioactive mediators, most notably histamine (Wang et al., 2021; Li et al., 2025). A study detected mRNA expression of TRPV1, TRPV2, and TRPV4 in peritoneal mast cells (PMCs) from mice using RT-PCR (Solís-López et al., 2017). In essence, TRPV1 is a cation channel that exhibits high permeability to Ca^2+^. Its activation leads to a significant influx of extracellular Ca^2+^, rapidly increasing intracellular Ca^2+^ concentration. This rise in Ca^2+^ is a crucial signal that triggers the release of mast cell granules, including histamine, through a process akin to cellular vomiting (Shim et al., 2007; Yu et al., 2019; Marek-Jozefowicz et al., 2023). Histamine induces itching by interacting with histamine H1 and H4 receptors (Akiyama and Carstens, 2013; Hashimoto et al., 2019). H1R and H4R on histaminergic neurons bind histamine and activate TRPV1 (Shim et al., 2007; Yosipovitch et al., 2018; Toyama et al., 2021). In herpes zoster, mast cell degranulation and the subsequent release of histamine and leukotrienes affect neutrophil adhesion and migration across the endothelium barrier, promote the polarization of pro-inflammatory M1 macrophages, exacerbate the process of inflammation, and ultimately lead to neuropathic pain (Gordon and Martinez, 2010; Kanashiro et al., 2020; Jiang et al., 2025). Clinical research shows that H3R and H4R antagonists can effectively alleviate pain hypersensitivity in neuropathological pain models (Borgonetti and Galeotti, 2022). The histamine system represents a promising target for the therapy of neurological pain, as supported by a growing body of research (Nuutinen and Panula, 2010; Marek-Jozefowicz et al., 2023). NGF is a protein secreted by various cells, including neurons and mast cells, that induces nerve growth. It binds to the protein tyrosine kinase receptor (TrKA), enhancing TRPV1 expression and promote its sensitization (Tan et al., 2021). Research shows that NGF is involved in the latent and reactivation phases of VZV *in vitro* (Baird et al., 2019). Wu et al. (2024) found that the plasma NGF levels in patients with shingles were significantly higher than those in the control group. NGF regulates neuronal growth, development, maintenance, and repair through TrkB-related pathways, suggesting a profound connection between NGF and HZN. Furthermore, NGF plays an important role in enhancing pain pathway sensitization and promoting inflammation, making it a key therapeutic target for the management of chronic pain diseases (Pulskamp et al., 2024). The mechanism by which NGF regulates TRPV1 have been extensively documented, demonstrating that NGF-induced pain sensitivity is partially mediated by TRPV1 activation (Eskander et al., 2015). Mast cells play a dual role in the NGF-TRPV1 feedback loop; by expressing the NGF receptor TrkA, mast cells can be directly influenced by NGF, leading to their degranulation and the release of various inflammatory mediators, including prostaglandins and histamine. Consequently, TRPV1 on neurons may become increasingly sensitive due to these mediators.

### 5.2 Regulation of TRPV1 on M1 Polarization of Macrophages

According to recent research, spinal microglia are primarily responsible for the development of postherpetic neuralgia, with activated glial cells playing a significant role in the central sensitization mechanisms underlying neuropathic pain (Kong et al., 2017; Fiore et al., 2023). Small glial cells, functioning as macrophages within the central nervous system, exert protective effects. Macrophages are essential for maintaining homeosis in the body. When the internal microenvironment changes, both the function and morphology of macrophages adapt accordingly, a process known as macrophage polarization. Undifferentiated macrophages polarize into M1 and M2 types in response to injury. Upon M1 polarization, DRGs release pro-inflammatory cytokines such as TNF-α, IL-1β, and IL-6. These cytokines facilitate viral replication, leading to neuronal necrosis and degeneration, ultimately resulting in neuropathic pain. This shows that inhibiting M1 polarization can effectively regulate neuroinflammatory responses and reduce pain sensitivity (Zhang et al., 2022; Tang et al., 2025b; Xu et al., 2025). Several inflammatory mediators are released during VZV infection due to inflammatory polarization of microglia. Among these, CGRP and substance P directly influence the state of microglial activation and may shift the protein balance towards the pro-inflammatory M1 phenotype during the acute inflammatory phase, potentially contributing to the development of HZN.

### 5.3 Bidirectional regulation of calcitonin gene-related peptide and TRPV1

Calcitonin gene-related peptide (CGRP) is a bioactive polypeptide composed of 37 amino acids released from sensory nerve endings and participating in multiple pathophysiological processes (Russo and Hay, 2023). CGRP mediates neurogenic inflammation and interacts with various inflammatory mediators and cytokines, contributing to the development of neuropathic pain (Hao et al., 2025; Maegawa et al., 2025; Sarkar et al., 2025). When TRPV1 neurons are activated, they transmit signals along their axons, which induce neurogenic reflexes that prompt cutaneous nerve terminals to release neuropeptides such as CGRP. This process triggers neurogenic inflammation and hyperalgesia in the skin (Choi and Di Nardo, 2018; Tarighi et al., 2019; Rivera-Mancilla et al., 2024). During this phase, the sustained release of neuropeptides and other mediators further sensitizes TRPV1 receptors on cutaneous nerve endings (Gouin et al., 2017; Marek-Jozefowicz et al., 2023). Devesa et al. (2014) found that CGRP enhances TRPV1 function by mobilizing TRPV1 to the neuronal cell membrane through a calcium-dependent molecular mechanism induced by sensitization, ultimately leading to hyperalgesia. Additionally, TRPV1 antagonists exert analgesic effects by inhibiting CGRP release and trigeminal nerve activation (Meents et al., 2015). Since activated TRPV1 enhances the release of CGRP and calcium-dependent substance P from peripheral nerve terminals, increased CGRP expression may facilitate the regeneration of injured peripheral neurons (Chen et al., 2019). Jiang et al. (2024) found that blocking CGRP release and TRPV1 activation can alleviate mechanical and thermal hyperalgesia in the DRG of mouse pain models, which is associated with the regulation of cytokines such as IL-1β, IL-6, and TNF-α.

## 6 Strategies for treating herpes zoster neuralgia by targeting TRPV1

### 6.1 TRPV1 agonists and antagonists: dual pharmacological strategies

TRPV1-targeted pharmacological strategies can be broadly divided into two categories: desensitizing agonists and channel-blocking antagonists. These two approaches differ fundamentally in their mechanisms of action but both aim to reduce pathological TRPV1 activity and nociceptive signaling in HZN (Szallasi and Blumberg, 1999; Iftinca et al., 2021). Research has revealed that TRPV1 modulates upstream regions in numerous pain transmission pathways, making it a novel therapeutic target for pain management (Bagood and Isseroff, 2021). Both agonists and antagonists of TRPV1 can produce analgesic effects (Gao et al., 2024, 2026). Postherpetic neuralgia has been shown to respond well to capsaicin and 8% capsaicin patches (Backonja et al., 2010; Yong et al., 2016; Bagood and Isseroff, 2021). Capsaicin is the first TRPV1 agonist to be identified; however, its application can lead to adverse reactions. Therefore, the study of TRPV1 antagonists may offer greater clinical applicability than agonists. TRPV1 antagonists inhibit pain by binding to TRPV1 channel protein, thereby preventing the channel from opening and blocking the inflow of Ca^2+^ triggered by stimuli such as capsaicin, heat or acid. This mechanism interrupts the transmission of harmful signals to the central nervous system, thereby achieving analgesic effects (Chen et al., 2024). Chen et al. (2025b) found that inhibiting the expression of TRPV1 can alleviate the pain associated with postherpetic neuralgia in mice. According to our team’s preliminary research, TRPV1 antagonists can lessen hyperalgesia by lowering TRPV1 expression in DRG neurons (Deng et al., 2025). The preclinical development of TRPV1 antagonists faces challenges primarily due to side effects arising from abnormalities in thermoregulation. TRPV1 channel antagonists can significantly affect body temperature, often resulting in hyperthermia; however, some may induce hypothermia or have no discernible effect (Huang et al., 2024). Despite extensive research into medication development, no TRPV1 antagonists have been authorized for clinical use to date (Tominaga and Iwata, 2025). Therefore, the objective of developing TRPV1 antagonists for pain therapy is to create medications that selectively block the activation of TRPV1 channels by pain inducers while preserving their activation by thermal stimulation (Chahl, 2024). During drug research, it is crucial to monitor subjects’ body temperature for abnormalities. The ongoing development of second-generation antagonists aims to mitigate side effects, such as high fever, while ensuring patient safety (Gavva, 2008). The upregulation of TRPV1 in HZN is spatially localized, specifically in DRG of affected nerve segments and the superficial dorsal horn of the spinal cord (Gao et al., 2024). This localization provides a pathological basis for selective inhibition. Systemic TRPV1 antagonists are known to have side effects related to thermoregulation. Further investigation is needed to determine whether these antagonists can be combined with peripheral local administration to circumvent thermoregulatory disorders and achieve a safe and innovative TRPV1-targeted therapy (Mobasheri et al., 2024). Together, these findings suggest that while TRPV1 represents a promising therapeutic target, successful drug development requires balancing analgesic efficacy with safety, particularly in relation to thermoregulation and sensory function.

### 6.2 New approaches to combination therapy

Combination therapy represents an emerging strategy to enhance the therapeutic efficacy of TRPV1-targeted interventions in HZN. The pathophysiological mechanisms underlying HZN are intricate, and therapies targeting a singular pathway often fail to yield satisfactory therapeutic outcomes. Combined treatment strategies have emerged as a pivotal approach in contemporary pain management, emphasizing the synergistic effects of pharmacological agents with diverse mechanisms to enhance therapeutic efficacy. This strategy not only minimizes the required dosages of individual medications but also mitigates the incidence of adverse reactions (Ji et al., 2016; Balanaser et al., 2023; Liu et al., 2026). TRPV1 antagonists alleviate pain and itching by inhibiting the receptor potential, thereby obstructing the transmission of nociceptive signals from the peripherals to the central nervous system (Zhu et al., 2023). Concurrently, these antagonists can diminish the side effects associated with opioid analgesics; their combined administration at peripheral primary sites has been shown to significantly alleviate neuropathic pain (Wang et al., 2022). The standard clinical treatment for HZN involves the use of antiviral drugs (such as acyclovir and valacyclovir) and first-line analgesics such as gabapentin and pregabalin (Guo et al., 2025), the use of TRPV1 antagonists in combination with these agents may represent a new therapeutic target for reducing nerve damage and alleviating HZN. A study conducted by Liu et al. demonstrated that ponesimod, a sphingosine-1-phosphate (S1P) antagonist, can normalize TRPV1 overexpression in the spinal cord, inhibit glial cell activation, reduce pro-inflammatory cytokines (IL-1β, TNF-α, IL-6), and inhibit MAPK phosphorylation. Notably, combining ponesimod with the TRPV1 antagonist AMG9810 enhanced the analgesic effect in rat studies targeting postherpetic neuralgia (Liu and Wang, 2025). Therefore, based on the involvement of NGF and CGRP highlighted in the aforementioned studies, exploring a combined inhibition strategy targeting TRPV1 represents a viable therapeutic avenue. Despite the challenges associated with the development of TRPV1 antagonists, the vast potential and prospects for treating intractable HZN disorders continue to be unveiled by a comprehensive understanding of TRPV1’s role and advancements in pharmaceutical chemistry.

### 6.3 Challenges and future directions in TRPV1-targeted drug development

Although considerable progress has been achieved in the development of TRPV1-targeted therapies for HZN, several challenges remain to be addressed (Maximiano et al., 2024). Systemic TRPV1 antagonists disrupt thermoregulation, and the widespread expression of TRPV1 in the peripheral and central nervous systems further increases the difficulty of selective targeting. The core to overcoming these challenges lies in distinguishing pathological and physiological TRPV1 signaling: allosteric modulators can inhibit pathological activation while retaining physiological functions such as thermoregulation (Aierken et al., 2021; Zhang et al., 2023); peripherally restricted antagonists (such as SB-705498) can avoid central adverse effects (Gunthorpe et al., 2007); combined with nano-targeted delivery systems, or combined treatment is expected to achieve precise intervention of lesions. In addition, in-depth analysis of the regulatory mechanism of TRPV1 in VZV-induced neuronal damage will provide a basis for the development of more disease-specific treatment strategies.

## 7 Summary

HZN is a direct manifestation of inflammation and damage caused by the virus invading the nerve. It is the primary symptom during the acute stage of the illness and may develop into chronic postherpetic neuralgia due to aberrant signal transmission and central sensitization after nerve damage. Current first-line therapies mainly target neuronal excitability or neurotransmitter release, but often fail to fully address the complex neuroinflammatory and neuroimmune mechanisms underlying HZN, highlighting the need for targets such as TRPV1 that act across multiple pathways. As a key analgesic target, TRPV1 is extensively involved in pain signal transmission and the regulation of neuroinflammation. It is distributed throughout the nervous system, immune cells, organ epithelial cells and skin keratinocytes. TRPV1 is capable of sensing various ions and regulating multiple cell signaling pathways. Although no study has fully elucidated the key role of TRPV1 in the human body, its function in different tissues and organs has been widely studied. TRPV1 is closely associated with DRG neurons, skin keratinocytes, and sensory nerve endings and plays a significant part in neuropathic pain and the immunological environment in postherpetic neuralgia. Further investigation into the molecular underpinnings of TRPV1 and the pathophysiology of related diseases can help aid in the design of safe and effective targeted inhibitory drugs. This progress will not only enhance the treatment of HZN, but also provide profound insights into the study of the entire field of neuropathological pain.

## Data Availability

The original contributions presented in the study are included in the article/supplementary material. Further inquiries can be directed to the corresponding author.
